# High-dose versus low-dose iron sucrose in individuals undergoing maintenance haemodialysis: a retrospective study

**DOI:** 10.1186/s12882-021-02570-0

**Published:** 2021-10-27

**Authors:** Luojin Liu, Huihui Cheng, Yukai Lv, Weiguang Yu, Qilong Liu, Yanqing Wu, Bo Xu

**Affiliations:** 1 Department of Nephrology, Shenzhen Longhua District Central Hospital, No. 187, Guanlan Avenue, Fumin Street, Longhua District, Shenzhen, 518110 China; 2 Department of Anesthesiology, Renmin Hospital of Wuhan University, Wuhan, 430060 Hubei China; 3 Department of Pediatrics, The First Affiliated Hospital, Sun Yat-sen University, No. 58, Zhongshan 2nd Road, Yuexiu District, Guangzhou, 510080 China; 4 Department of Orthopaedics, The First Affiliated Hospital, Sun Yat-sen University, No. 58, Zhongshan 2nd Road, Yuexiu District, Guangzhou, 510080 China; 5 Department of Gastrointestinal Surgery, The First Affiliated Hospital, Sun Yat-sen University, No. 58, Zhongshan 2nd Road, Yuexiu District, Guangzhou, 510080 China; 6 Department of Thyroid Breast Surgery, The First Affiliated Hospital, Sun Yat-sen University, No. 58, Zhongshan 2nd Road, Yuexiu District, Guangzhou, 510080 China; 7 Department of Cardiothoracic Surgery, The First Affiliated Hospital, Sun Yat-sen University, No. 58, Zhongshan 2nd Road, Yuexiu District, Guangzhou, 510080 China

**Keywords:** Haemodialysis, Iron, Maintenance, Event, Infection

## Abstract

**Background:**

Intravenous iron sucrose is becoming a prevailing treatment for individuals undergoing maintenance haemodialysis, but comparisons of dosing regimens are lacking. The aim of this retrospective review was to evaluate the safety and efficacy of proactively administered high-dose iron sucrose versus reactively administered low-dose iron sucrose in patients undergoing maintenance haemodialysis.

**Methods:**

We analysed the data of 1500 individuals with maintenance haemodialysis who were treated with either high-dose iron sucrose that was proactively administered (Group HD) or low-dose iron sucrose that was reactively administered (Group LD) at the First Affiliated Hospital of Chongqing Medical University from Jan 1, 2008, to Dec 31, 2020. The primary endpoints were the cumulative doses of iron and erythropoiesis-stimulating agent; the secondary endpoints were the events of nonfatal myocardial infarction, nonfatal stroke, hospitalization for heart failure, infection rate, and death from any cause.

**Results:**

Of the 2124 individuals, 624 individuals were excluded because they met one or more of the exclusion criteria, thus resulting in 1500 individuals who were eligible for inclusion in the study (Group HD, *n* = 760 and Group LD, *n* = 740). The median follow-up for the two cohorts was 32 months (range: 25–36). A significant median difference was detected in the monthly iron dose between the groups (1121 mg [range: 800–1274] in the HD group vs. 366 mg [range: 310–690] in the LD group; *p* < 0.05). The median dose of an erythropoiesis-stimulating agent was 26,323 IU/month (range: 17,596-44,712) in the HD group and 37,934 IU/month (range: 22,402-59,380) in the LD group (median difference: − 7901 IU/month; 95% CI: − 9632--5013; *p* = 0.000). A significant difference was detected in the secondary endpoints (266 events in 320 cases in the HD group vs. 344 events in 385 cases in the LD group) (HR: 0.62; 95% CI: 0.51–0.79; *p* < 0.001). A significant difference was not observed in death from any cause (HR: 0.57; 95% CI: 0.48–1.00; *p* = 0.361).

**Conclusions:**

For individuals undergoing maintenance haemodialysis, high-dose iron sucrose that was proactively administered may be superior to low-dose iron sucrose that was reactively administered with low doses of erythropoiesis-stimulating agent.

## Background

Renal failure is generally considered to be a discouraging disease affecting patients throughout the world, due to its association with increased rates of mortality [1–4]. The incidences of renal failure and associated mortality in China have been increasing since 2009 [1, 4, 5]. The management of renal failure remains challenging, even though early-stage renal failure can potentially be cured [6–8]. For late-stage renal failure, the general recommendation for patients is maintenance haemodialysis [9–12], and it may be a frequently used option regardless of a renal transplant [11]. Although recent clinical outcomes for such cases have improved, the optimal regimen for maintenance haemodialysis still has some details to be discussed [9, 11, 13].

Patients with maintenance haemodialysis tend to suffer from negative iron balance, which is primarily attributed to reduced iron absorption and increased iron loss [14, 15]. Intravenous iron sucrose has been considered as a standard processing scheme, as has been reported in prior studies [9, 15, 16]. Increasing doses of iron sucrose can counteract exposure to erythropoiesis-stimulating agents, with the aim of decreasing the potential adverse events (AEs) that are associated with these agents, especially infection and cardiovascular events [9, 14, 17, 18].

Ambiguity has continued to arise concerning the subject of the optimal utilization of iron, even though high-quality evidence related to the use of erythropoietin stimulating agent and haemoglobin targets in patients with maintenance haemodialysis has been documented [9, 16]. Moreover, the evidence-based assessment of the utilization of proactively administered high-dose iron sucrose in these patients is exceedingly limited [9, 19]. Thus, there is a continuing debate about the benefits of proactively administered high-dose iron sucrose versus reactively administered low-dose iron sucrose [9, 14, 19]. Herein, we aimed to confirm whether patients undergoing maintenance haemodialysis who were treated with proactively administered high-dose iron sucrose had greater clinical benefits than those patients receiving reactively administered low-dose iron sucrose.

## Methods

### Study design and patient eligibility

Data for 2124 patients with maintenance haemodialysis who were treated with proactively administered high-dose iron sucrose or reactively administered low-dose iron sucrose were retrospectively identified and retrieved from three medical centers of the First Affiliated Hospital, Sun Yat-sen University, Shenzhen Longhua District Central Hospital, and Renmin Hospital of Wuhan University from Jan 1, 2008, to Dec 31, 2020. The dialysis regimen is based on the Chinese version of the clinical practice guidelines for hemodialysis adequacy. Each medical centre receives an average of 80–100 new patients per year. About a third of patients receive long-term dialysis. Dialysis services at these centres are mainly covered by the national health insurance, and very little by patients themselves. All of the demographic data, iron regimens, iron doses, the doses of erythropoiesis-stimulating agents, the events of nonfatal myocardial infarction, nonfatal stroke, hospitalizations for heart failure, infection rates, and deaths from any cause were obtained by three co-authors from the medical charts and the follow-up data. The eligible criteria included patients aged ≥18 years; patients who were definitively diagnosed with end-stage kidney disease; patients with a ferritin concentration < 400 μg/L; patients with a transferrin saturation < 30%; and patients who were receiving an erythropoiesis-stimulating agent. The following key exclusion criteria were used: patients lacking baseline data (i.e., losses to follow-up and withdrawals of consent); patients with allergic reactions to iron sucrose; patients with peritoneal dialysis; patients who experienced discontinuations that were instigated by non-drug factors in the high-dose or low-dose regimens; patients with kidney transplantations; patients with serious digestive disorders (i.e., ulcerative colitis, pancreatitis, and choledocholithiasis); patients with hypersplenism; patients with severe infectious diseases (i.e., human immunodeficiency virus, acute respiratory distress syndrome, septicaemia, or septicopyemia); patients with tumours; patients with coagulation disorders; patients with cognition impairments; or patients with mental disorders.

### Study design and treatment

We conducted this retrospective, multicentre review in which eligible patients underwent either proactively administered high-dose iron sucrose (Group HD at 400 mg/month; once the ferritin concentration of ≥700 μg/L or a transferrin saturation of ≥40% occurred, the intravenous iron sucrose was terminated) [9] or reactively administered low-dose iron sucrose (Group LD at 0–400 mg as required to sustain a target ferritin concentration of ≥200 μg/L and a transferrin saturation of ≥20%) [9, 20]. The maintenance of a haemoglobin level of 10–12 g/dL was the ultimate goal of the erythropoiesis-stimulating agent [20].

### Outcomes and assessments

The primary endpoints were the cumulative doses of iron and erythropoiesis-stimulating agent. The secondary endpoints were the events of nonfatal myocardial infarction, nonfatal stroke, hospitalization for heart failure, infection rate, and death from any cause. The measurements of haemoglobin levels, serum ferritin concentrations, and transferrin saturation levels were repeated at 30-day intervals. Patient’s comorbidity and secondary endpoint measures were in accordance with International Classification of Diseases, 9th Revision. Serious AEs were collected based on previous descriptions [9]. Baseline variables, treatment histories, and the dates of initial administrations of intravenous iron sucrose were collected, along with the final follow-up.

### Statistical analysis

Baseline variables are presented as numbers and percentages, standard deviations (SD), and interquartile ranges. We used chi-square tests for the analysis of the categorical variables and either Student’s t-tests (normally distributed data) or Mann-Whitney U tests (non-normally distributed data) for the analysis of the continuous variables. The time-to-first event analyses were performed by using cause-specific Cox proportional hazard models, regardless of the durations or doses of the intravenous iron sucrose. The median follow-up was assessed by using the reverse Kaplan-Meier method. Hazard ratios (HRs) were estimated by using the Cox proportional hazard model with a 95% confidence interval (95% CI). Between-group comparisons of the cumulative doses of iron sucrose were performed by using Wilcoxon rank-sum tests. All of the statistical analyses were performed by using SPSS 26.0 (IBM, Inc., NY, USA). A two-sided *p* value < 0.05 was regarded as being statistically significant.

## Results

### Baseline characteristics

**Fig. 1:**
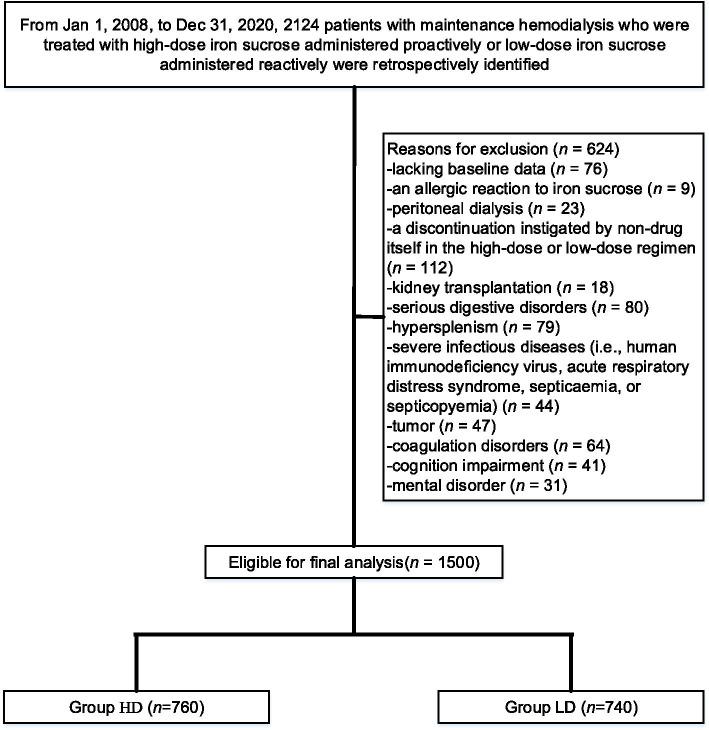
Flow diagram exhibiting the methods applied to identify objects to evaluate the safety and efficacy of high-dose iron sucrose administered proactively versus low-dose iron sucrose administered reactively in patients undergoing maintenance hemodialysis

We identified 2124 patients with maintenance haemodialysis, of whom 624 individuals were excluded because they met one or more of the exclusion criteria, thus resulting in 1500 individuals who were eligible for inclusion in the study. Of these patients, 760 received high-dose iron sucrose that was proactively administered, and 740 received low-dose iron sucrose that was reactively administered, as is shown in Fig. 1. Table 1 summarizes the patient characteristics that were well balanced between the two cohorts. The median age was 54 years (range: 42–64) in the HD group and 54 years (range: 40–66) in the LD group. There were 420 men and 340 women in the HD group and 414 men and 326 women in the LD group (*p* = 0.213). The median duration of dialysis was 6.2 months (range: 3.2–9.7) in the HD group and 6.1 months (range: 3.1–9.5) in the LD group. Dialysis catheter and arteriovenous fistula or graft procedures represented 37.4 and 62.6% in the HD group, respectively, versus 39.2 and 60.8% in the LD group, respectively (*p* = 0.468). Cardiovascular diseases mainly involved hypertension in 48.3% of patients and hyperlipidaemia in 24.5% of patients receiving high-dose iron sucrose that was proactively administered, compared to hypertension in 46.2% of patients and hyperlipidaemia in 26.5% of patients receiving low-dose iron sucrose that was reactively administered (*p* < 0.05). The median follow-up for the two cohorts was 32 months (range: 25–36). The median number of administration times was 32 (range: 25–36) for patients receiving proactively administered high-dose iron sucrose and 16 (range: 12–18) for those patients who received reactively administered low-dose iron sucrose. Sixty-seven (9.1%) patients who were treated with reactively administered low-dose iron sucrose were converted to proactively administered high-dose iron sucrose prior to death.

**Table 1:** Patient demographics between groups

Variable	HD (*n* = 760)	LD (*n* = 740)	*P*-value
Age^d^ (years)	54(42–64)	54(40–66)	0.174^*a*^
Sex male/female	420/340	414/326	0.213^*b*^
Median duration of dialysis (months)	6.2(3.2–9.7)	6.1(3.1–9.5)	0.151^*a*^
BMI^d^, kg/m^2^	26.2(21.1–31.4)	26.3(21.6–31.8)	0.112^*a*^
Blood pressure^de^ (mmHg)			0.919^*c*^
Systolic	149(120–174)	148(115–182)	
Diastolic	72(59–90)	73(60–89)	
Hemoglobin^d^ (g/dl)	10.4(8.5–12.1)	10.8(8.1–12.7)	0.346^*a*^
Median serum ferritin concentration (μg/l)	221(126–297)	220(124–298)	0.109^*a*^
Median transferrin saturation (%)	21 (14–25)	21 (13–25)	0.824^*a*^
Median C-reactive protein level (mg/l)	6.3 (3.1–14.6)	6.4 (3.2–15.8)	0.472^*a*^
Median dose of ESA (IU/wk)	9000 (4000–11,000)	9000 (4000–11,000)	0.163^*a*^
Vascular access, n (%)			0.468^*c*^
Dialysis catheter	284(37.4)	290(39.2)	
Arteriovenous fistula or graft	476(62.6)	450(60.8)	
Cardiovascular disease, n (%)			0.537^*c*^
Prior myocardial infarction	64(8.4)	74(10.0)	
Hyperlipidemia	186(24.5)	196(26.5)	
Atrial fibrillation	76(10.0)	53(7.2)	
Hypertension	367(48.3)	342(46.2)	
Heart failure	67(8.8)	75(10.1)	
Diabetes, n (%)	216 (28.4)	202 (27.3)	
Primary cause of kidney failure, n (%)			0.200^*c*^
Diabetic nephropathy	221(11)	200(27.0)	
Glomerular disease	143(19)	141(19.0)	
Hypertension	287(26)	270(36.5)	
Tubulointerstitial disease^f^	65(15)	76(10.3)	
Polycystic kidney disease	30(21)	33(4.5)	
Unclear	14(8)	20(2.7)	

*HD* High-dose iron sucrose administered proactively, *LD* Low-dose iron sucrose administered reactively, *BMI* Body mass index, *ESA* Erythropoiesis-stimulating agent

^a^Independent-Samples t-test

^b^Chi-square test

^c^Mann-Whitney U test

^d^IQR represents interquartile range (25th to 75th percentile)

^e^Measurements were done prior to hemodialysis

^f^Tubulointerstitial disease included pyelonephritis, reflux nephropathy, and obstructive uropathy

### Primary endpoints

**Fig. 2:**
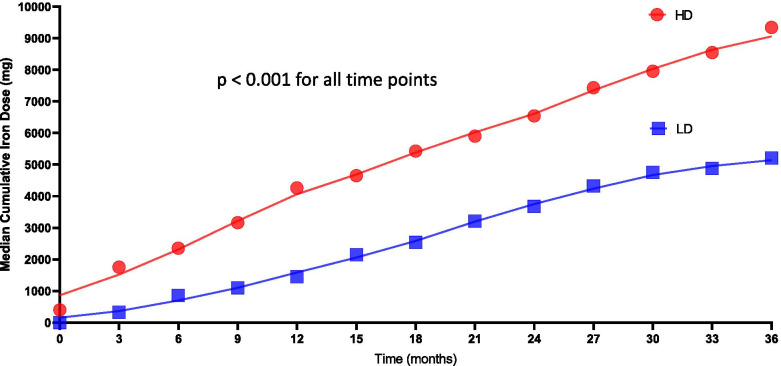
Iron administration over time. The mean cumulative doses of intravenous iron that were received by the patients in both groups were presented over time. At all the time points, HD-treated patients had greater cumulative doses of iron than LD-treated patients (*p* < 0.001 for all time points)

The HD-treated cohort had greater cumulative doses of intravenous iron than the LD-treated cohort, as presented in Fig. 2. At the 12th month, the HD-treated cohort had received more iron than the LD-treated cohort. The median monthly dose of intravenous iron was 1121 mg (range: 800–1274) in the HD group and 366 mg (range: 310–690) in the LD group; additionally, the median difference in the monthly iron dose was 755 mg (95% CI: 681–877). The HD-treated cohort had significantly increased ferritin concentrations and transferrin saturation levels compared with the LD-treated cohort (*p* < 0.05).

**Fig. 3:**
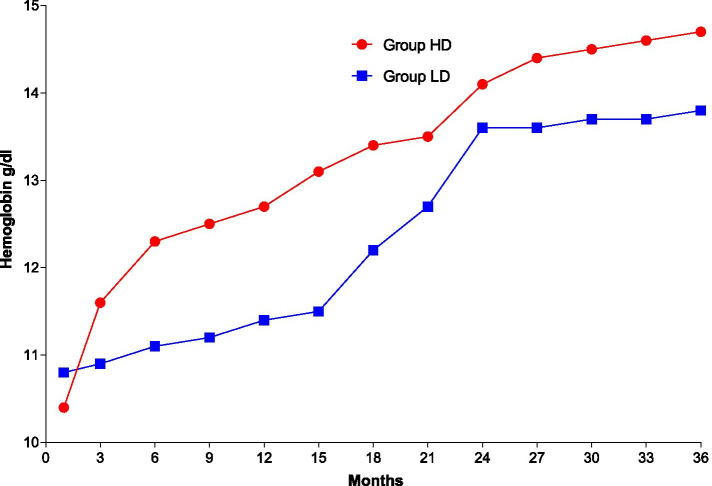
Hemoglobin levels throughout the follow-up period. During the follow-up period, the hemoglobin levels of both groups were significantly improved compared with baseline. Group HD maintained a higher level at the final follow-up compared with Group LD(*p* = 0.014)

A lower cumulative dose of erythropoiesis-stimulating agent was observed in the HD-treated cohort than in the LD-treated cohort at each follow-up. The median monthly dose of erythropoiesis-stimulating agent was lower in the HD-treated cohort (26,323 IU/month; range: 17,596-44,712) than in the LD-treated cohort (37,934 IU/month; range: 22,402-59,380) (median difference: − 7901 IU/month; 95% CI: − 9632--5013; *p* = 0.000). Although the haemoglobin levels of both cohorts increased from the baseline level over time, they increased more quickly in the HD-treated cohort than in the LD-treated cohort. At the final follow-up, the hemoglobin level in the HD group was significantly higher than in the LD group (*p* = 0.014), as shown in Fig. 3.

### Secondary endpoints

**Fig. 4:**
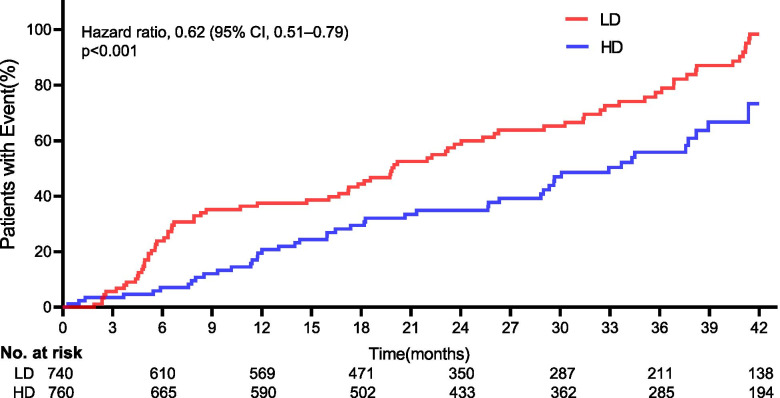
Cumulative incidence of the cumulative events. Kaplan-Meier curves showed a significant separation (Hazard ratio, 0.62, 95%CI, 0.51–0.79; *p* = 0.361). *The hazard ratio was calculated using a Cox proportional hazards model, with the age, median duration of dialysis, body mass index, Blood pressure, vascular access, primary cause of kidney failure, and median C-reactive protein level used as covariates and therapy as the time-dependent factor

**Fig. 5:**
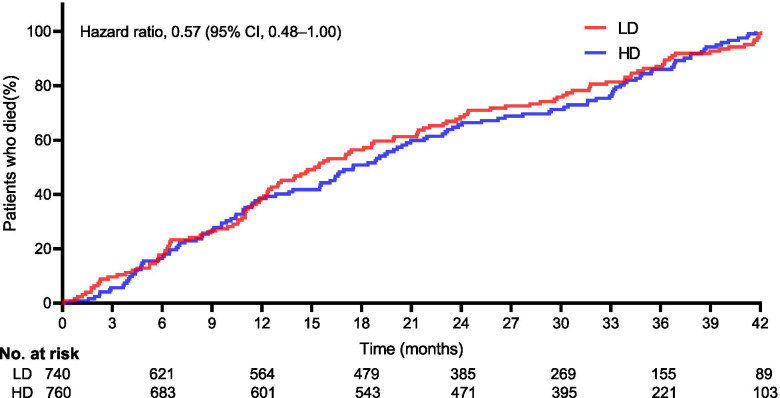
Cumulative incidence of the death from any cause. Kaplan-Meier curves did not show a significant separation (Hazard ratio, 0.57, 95%CI, 0.48–1.00; *p* = 0.361). *The hazard ratio was calculated using a Cox proportional hazards model, with the age, median duration of dialysis, body mass index, Blood pressure, vascular access, primary cause of kidney failure, and median C-reactive protein level used as covariates and therapy as the time-dependent factor

Two hundred and sixty-six secondary endpoint events were observed in 320 cases (42.1%) in the HD cohort compared to 344 in 385 cases (52.0%) in the LD cohort (HR: 0.62; 95% CI: 0.51–0.79; *p* < 0.001), as shown in Table 2 and Fig. 4. The rate of AEs, nonfatal myocardial infarctions, nonfatal strokes, hospitalizations for heart failure, infections or infestations, and death from any cause was lower in the HD cohort than in the LD cohort. A significant difference was not observed for death from any cause (HR: 0.57; 95% CI: 0.48–1.00; *p* = 0.361), as shown in Fig. 5. Significant differences were observed in regard to the total AEs, nonfatal myocardial infarctions, and hospitalizations for heart failure (all *p* < 0.05).

**Table 2:** Comparison of the incidence of key drug-related AEs between groups at final follow-up

Event	HD (*n* = 760)	LD (*n* = 740)	*P*-value
Total AEs, n (%)	266(35.0)	344(46.5)	0.001*
Nonfatal myocardial infarction, n (%)	48(6.3)	77(10.4)	0.004
Nonfatal stroke, n (%)	55(7.2)	62(8.4)	0.410
Hospitalization for heart failure, n (%)	41(5.4)	74(10.0)	0.001
Infection or infestation, n (%)	65(8.6)	69(9.3)	0.600
Death from any cause, n (%)	57(7.5)	62(8.4)	0.223

*AEs* Adverse events, *HD* High-dose iron sucrose administered proactively, *LD* Low-dose iron sucrose administered reactively

^*^Statistically significant values

## Discussion

This current study showed that patients with maintenance haemodialysis receiving low-dose intravenous iron that was reactively administered do not seem to exhibit more clinical benefits from intravenous iron than those patients receiving high-dose intravenous iron that was proactively administered, regardless of the treatment costs. The superiority of the high-dose intravenous iron that was proactively administered over the low-dose intravenous iron that was reactively administered in this setting tended to be remarkable, and the high-dose intravenous iron that was proactively administered was associated with a lower rate of nonfatal myocardial infarctions, hospitalizations for heart failure, or total AEs, when compared with the low-dose intravenous iron that was reactively administered. To the best of our knowledge, this retrospective review is the largest study to date examining Chinese patients undergoing maintenance haemodialysis.

Patients who experienced proactively administered high-dose intravenous iron were less likely to suffer nonfatal myocardial infarctions than those patients who underwent reactively administered low-dose intravenous iron. It is possible that a high-dose intravenous injection of iron in these cases with iron deficiency can reduce the risk of cardiovascular AEs [14]. Additionally, a high-dose intravenous iron regimen tends to decrease the incidence of hospitalization for heart failure. Although the intravenous iron regimen has been observed to result in a low rate of cardiovascular AEs in previous studies [9, 14] involving patients with circulatory failure, to date, such a scenario has not been detected in patients undergoing maintenance haemodialysis. Additionally, patients who underwent a high-dose intravenous iron regimen failed to have more blood transfusions and higher doses of erythropoiesis-stimulating agents than those who underwent a low-dose intravenous iron regimen. The findings from the present study were consistent with a multicentre, open-label trial [9], which showed that a high-dose intravenous iron regimen reduces the amount of erythropoiesis-stimulating agent in patients undergoing haemodialysis.

Consistent with the results from this study, a previous multicentre, open-label, blinded endpoint, randomized controlled trial [21] showed that a proactive high-dose intravenous iron regimen had noteworthy advantages, in terms of first-appearing nonfatal myocardial infarctions, hospitalizations for heart failure, and reduced monthly doses of an erythropoiesis stimulating agent. In the trial, 2589 haemodialysis patients from 50 institutions in the United Kingdom were enrolled and were followed for 4.5 years (median: 2.1 years), of whom 2141 (83%) were randomized to undergo a proactive high-dose intravenous iron regimen (400 mg/month, unless ferritin > 700 μg/L and/or transferrin saturation ≥ 40%) or a reactive low-dose intravenous iron regimen that maintained patients near the lowest acceptable iron limits (iron sucrose being administered if ferritin < 200 μg/L or transferrin saturation < 20%). Patients underwent a median iron dose of 264 mg/month in the high-dose group versus 145 mg/month in the low-dose group.

Although the effectiveness of erythropoiesis-stimulating agents has been verified in patients using maintenance haemodialysis [9, 16], data in the patient population remain limited. In a recent randomized study [22], 200 chronic haemodialysis patients with functional iron deficiency anaemia in Thailand were included. These patients with transferrin saturation of < 30% and serum ferritin of 200–400 ng/mL were randomized 1:1 to maintain serum ferritin at either 200–400 ng/mL or 600–700 ng/mL. This study demonstrated that the maintenance of a high serum ferritin level via intravenous iron administration at 200 mg/month can result in a reduced dose of an erythropoiesis stimulating agent in these patients. These different treatment regimens have similar clinical benefits in decreasing the use of erythropoietin doses. Although previous reports [9, 19, 23] have raised the concern that monthly doses of < 400 mg of intravenous iron tended to be associated with good clinical outcomes, the high-dose intravenous iron level of ≥400 mg/month that is proactively administered can increase the saturation of serum ferritin and transferrin and reduce the dependence on erythropoiesis stimulating agents [24].

Given that erythropoiesis-stimulating agents can elevate haemoglobin levels [18, 25], concerns regarding the safety of the high-dose use of erythropoiesis-stimulating agents have been raised, as cardiovascular toxic effects are associated with high haemoglobin levels [9, 14, 19, 24]. At present, there remains a paucity of data regarding the maintenance of target haemoglobin levels. Although there is a recognition of a clear separation of haemoglobin level curves, in cases of favouring the continuation of a high-dose intravenous iron regimen, the continuation of a high-dose intravenous iron regimen beyond the limit level of haemoglobin has failed to produce favourable outcomes [9]. Furthermore, the improvements in the clinical benefits appear to be small for haemodialysis patients and tend to be associated with the timing of haemoglobin assessments [9, 14]. However, there are frequent debates concerning the influence of intravenous iron doses [15]. Furthermore, there is a gap in the understanding of different intravenous iron regimens, which needs to be addressed for haemodialysis patients.

Several drawbacks should be acknowledged in the present study. It was a retrospective study with some problems inherent with this methodology. Retrospective data collection, patient heterogeneity, and variable regimen due to a time span of more than 10 years limit the veracity of the data and may lead to the possibility of indication and clinician bias. In an effort to address these concerns, a large number of hemodialysis patients with similar baseline data were included and it was possible to assess the safety and efficacy of proactively administered high-dose iron sucrose versus reactively administered low-dose iron sucrose in patients undergoing maintenance haemodialysis with widely used endpoint measures. This study included a population of patients with a median age of 54 years (range: 40–66) undergoing maintenance haemodialysis; therefore, it is unclear as to whether the findings can be translated to patients with a median age of less than or more than 40 years. Additionally, some potential variables (i.e., pneumonia, bacteremia, or tuberculosis) were not stratified in this study. Furthermore, the present study involved a subset of patients with high-frequency (more than 4 administrations per month) haemodialysis, although the intravenous iron regimen was invariable, and the baseline data were similar between the two cohorts. In view of the low endpoint event rate (specifically in regard to the low recurrence event rate), CIs tended to be broad; hence, the estimated benefit of the high-dose intravenous iron regimen at individual endpoint event values should be clarified with caution.

## Conclusion

The results reported in this study may support the growing body of evidence that high-dose iron sucrose that is proactively administered may be superior to low-dose iron sucrose that is reactively administered with low doses of erythropoiesis-stimulating agent for Chinese patients undergoing maintenance haemodialysis. However, the use of high-dose iron sucrose that is proactively administered may require systematic assessment of the balance between intended clinical benefits and indefinite risks of toxicities, and the potential effects of high-dose iron sucrose that is proactively administered on the clinical benefits remain unknown. It remains to be demonstrated that fewer AEs and more clinical benefits would actually result in different clinical decisions concerning treatment. Further validation with prospective data collection will be necessary.

## Data Availability

The datasets used and analyzed in this study are available from the corresponding authors on reasonable request.

## Abbreviations

HD: High-dose iron sucrose administered proactively
LD: Low-dose iron sucrose administered reactively
AEs: Adverse events
HR: Hazard ratio
CI: Confidence interval
